# 865 12 Year Experience of Inhalation Injuries from House and Structure Fires

**DOI:** 10.1093/jbcr/iraf019.396

**Published:** 2025-04-01

**Authors:** Christopher Fedor, Mare Kaulakis, Hilary Liu, José Arellano, Garth Elias, Alain Corcos, Matthew Siedsma, Jenny Ziembicki, Francesco Egro

**Affiliations:** University of Pittsburgh School of Medicine; University of Pittsburgh School of Medicine; University of Pittsburgh Medical Center; University of Pittsburgh Medical Center; University of Pittsburgh Medical Center; University of Pittsburgh Medical Center; University of Pittsburgh Medical Center; University of Pittsburgh Medical Center; University of Pittsburgh Medical Center

## Abstract

**Introduction:**

Inhalation injuries from house and structure fires can pose significant risks, including infection, inflammation, and multi-organ dysfunction. Because inhalation injuries are an understudied aspect of acute burn care, the aim of this study was to identify and analyze salient factors affecting clinical outcomes for this vulnerable patient population.

**Methods:**

We performed a 12-year retrospective study (January 2012 to January 2024) of house-fire patients treated at an ABA-certified burn center. Flash burns from smoking on home oxygen therapy were excluded as they do not maintain the characteristics of true inhalation injuries. The severity of inhalation injury was assessed using the Abbreviated Injury Score (AIS) following fiberoptic bronchoscopy. Data involving complications, ventilator days, and length of stay were collected.

**Results:**

Of 462 patients in house or structure fires, 222 (48%) underwent diagnostic bronchoscopy. 184 of these had findings consistent with inhalation injury (75 mild, 79 moderate, and 30 severe). The cohort’s average age was 54 ± 19 years, with a male-to-female ratio of 1.3. Most injuries occurred in November through January. 31.5% patients (n=58) were intubated at the scene, and 64.7% (n=119) upon hospital arrival. 33.2% (n=61) initially presented at an outside hospital before being transferred to our burn unit. The median TBSA of cutaneous burns was 1% (IQR: 0-14), and median carboxyhemoglobin was 11.8 (IQR: 5.9-26.8). 50 patients (27.2%) underwent excision and grafting, with a median time to graft of 5 days (IQR: 3-6). The median ventilator time was 3 days (IQR: 1–9.5), and the median hospital stay was 10.5 days (IQR: 3.5–21). Pneumonia was the most common complication (41.3%). 26 patients (14.1%) developed ARDS, and there were 34 deaths within one month (18.5%). After adjusting for age, BMI, TBSA, and carbon monoxide poisoning, patients with moderate or severe injuries were hospitalized for an additional 6.8 days (p = 0.008) and 5.7 days (p = 0.066), respectively, compared to those with mild injuries. Moderate and severe injuries also resulted in 3.4 days (p = 0.048) and 4.6 days (p = 0.044) longer on a ventilator than mild injuries.

**Conclusions:**

Most patients suffering inhalation injuries have carbon monoxide poisoning and minimal cutaneous burns. While carbon monoxide poisoning was not an accurate predictor of ventilator days nor hospital length of stay, the extent of visible airway injury seen on bronchoscopy correlated significantly with these outcomes. These findings underscore the importance of thorough and accurate use of bronchoscopy evaluations in the acute period for prognostication and management planning.

**Applicability of Research to Practice:**

Studying hospital outcomes for inhalation injuries enhances prognostication, multidisciplinary care, and the identification of at-risk populations. This research also helps recognize clinical patterns and risk factors associated with poorer outcomes.

**Funding for the Study:**

N/A

